# The effect of sample attrition in the EU Statistics on Income and Living Conditions on the estimates of Eurostat’s Healthy Life Years

**DOI:** 10.1093/eurpub/ckad069

**Published:** 2023-04-24

**Authors:** Magdalena M Muszyńska-Spielauer, Martin Spielauer

**Affiliations:** Vienna Institute of Demography (OeAW), Wittgenstein Centre for Demography and Global Human Capital (IIASA, OeAW, University of Vienna), Vienna, Austria; Department of Public Finance and Infrastructure Policy, Institute of Spatial Planning, TU Wien, Vienna, Austria; Austrian Institute of Economic Research, Vienna, Austria

## Abstract

Eurostat’s official Healthy Life Years (HLY) estimates are based on European Union Statistics on Income and Living Conditions (EU-SILC) cross-sectional data. As EU-SILC has a rotational sample design, the largest part of the samples are longitudinal, health-related attrition constituting a potential source of bias of these estimates. Bland-Altman plots assessing the agreement between pairs of HLY based on total and new rotational, representative samples demonstrated no significant, systematic attrition-related bias. However, the wide limits of agreement indicate considerable uncertainty, larger than accounted for in the confidence intervals of HLY estimates.

## Introduction

Longitudinal samples are subject to attrition, which is the dropout of participants between subsequent interviews. Attrition reduces the sample size and can lead to erroneous results, hence conclusions, because it may bias the sample by changing its composition so that it is no longer representative of the study population. In particular, attrition will bias results if the characteristics being studied, or other related characteristics, also influence the probability of dropping out from the sample. This case is apparent in health studies, where the outcome variable, health status, and health-related characteristics, i.e. sex, old age, marital status and educational attainment, are known determinants of sample attrition.^1^^,^^2^ The problem of sample attrition and its potential bias in measuring phenomena and their relationships is well recognized in longitudinal studies, but rarely in cross-sectional studies based on longitudinal samples. Attrition affects measures based on cross-sectional data sets of the Income and Living Conditions Survey (EU-SILC), where three out of four sub-samples are longitudinal. *Healthy Life Years* (HLY), published by Eurostat and constituting the official indicator for monitoring the health status of Europeans, is an example of such a measure. This short report aims to assess the extent to which Eurostat’s HLY is affected by attrition.

## Methods

We replicate the official Eurostat’s HLY statistics.^3^ In HLY, a healthy state is defined by the absence of long-term limitations in activities of daily living according to the global activity limitation indicator. HLY is estimated using the Sullivan method, which redistributes years lived at a given age from a life table into the healthy and unhealthy parts, according to the prevalence of health-related limitations. The prevalence of health-related limitations comes from 309 country-period cross-sectional datasets (up to 13 waves for 26 countries) of the publicly available EU-SILC datasets^4^ for 2007–19. In estimating prevalence, we apply personal weights in EU-SILC. Life years are taken from the Eurostat life tables.^5^

A limitation of the method is that the gap between the two HLYs depends on the number of years lived, which is derived from the official life tables. Therefore, in comparative studies, a larger gap between the HLYs does not necessarily mean a larger effect of attrition on the prevalence statistics, but a higher number of years lived due to lower mortality.

Attrition bias in cross-sectional health statistics in EU-SILC is assessed by agreement between HLYs estimated from total samples with those derived solely from new rotational sub-samples. The new rotational sub-samples are newly drawn, representative samples, consisting of respondents who receive the questionnaire simultaneously as the respondents from the remaining longitudinal sub-samples. The advantage of using information from such an additional sub-sample is that it requires fewer assumptions about missing data than other methods.^6^ In SILC, apart from stabilizing the number of observations, the rotational sub-samples are explicitly drawn to provide a means of assessing attrition bias.^7^ The sequence number identifying a sub-sample was identified on the basis of country-specific survey documentation (see Supplementary material). The statistics are estimated at ages 0, 50 and 65, as the effect of health-related attrition on the gap is likely to be age-specific: Previous studies have shown that, at younger age groups those who are healthy are more likely to attrit and the opposite is true at older ages.^8^

Agreement between the two HLYs, which represent the comparability of results obtained from the two measurement methods, is assessed using Bland–Altman (B&A) plots with 95% limits of agreement (LoA).^9^ A detailed description of the method can be found in the Supplementary material. The method requires normality of the distributions of differences between the two measurements, which was verified by the Shapiro–Wilk test (*P* < 0.01).

Finally, the uncertainty of the Eurostat estimates of HLYs related to attrition is discussed by contrasting the confidence intervals (CIs) for total-sample HLYs with the limits of agreement of the B&A plots. As Eurostat does not publish CIs, we estimated them following the guidelines of Jagger *et al*.^10^

## Results

**Figure 1.**
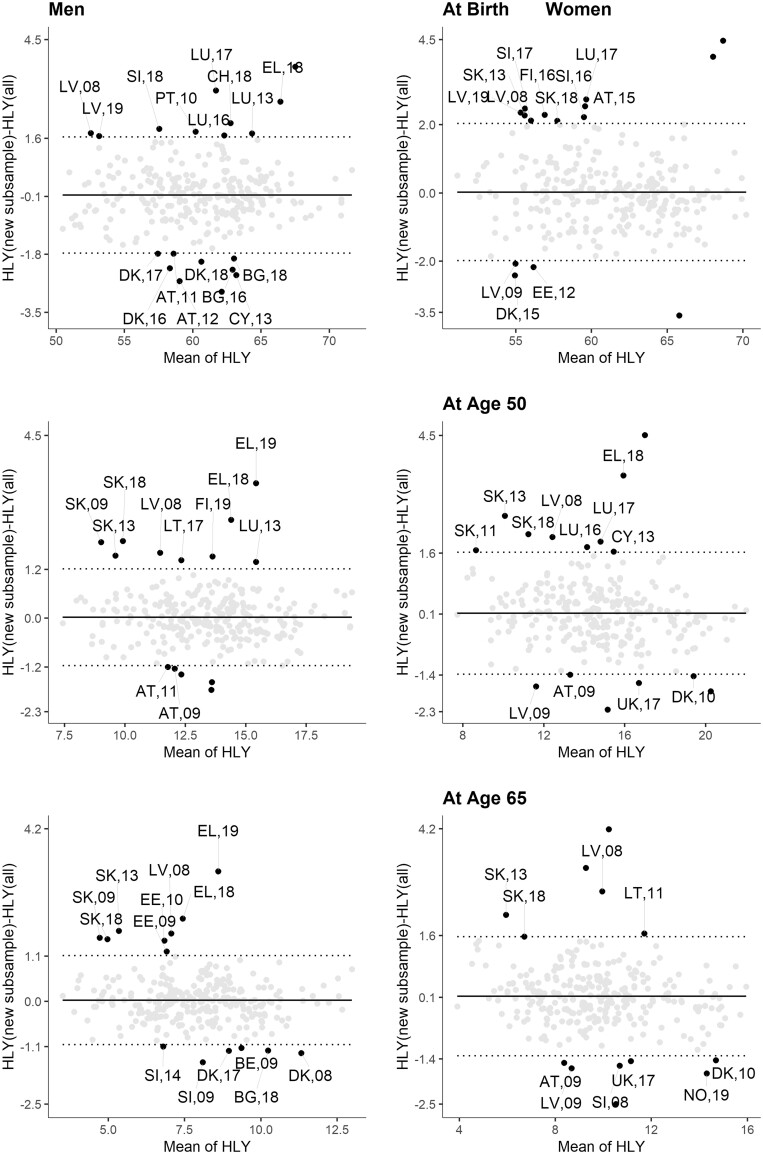
Bland–Altman plots of Healthy Life Years (HLY) based on total cross-sectional and new rotational EU-SILC samples. Notes: Horizontal solid line indicates the mean differences between the two HLYs. The upper and lower dotted lines are 95% confidence intervals for the differences. Source: Authors’ estimations based on ref.^4^

Figure 1 presents B&A plots for HLYs by sex and ages 0, 50 and 65 years, with 309 country-period observations. A positive value of the difference between HLYs (on the *Y* axis) indicates that the HLY based on the total cross-sectional sample is overestimated, compared to the HLY derived from a representative, new sub-sample. This means that those with health-related limitations are more likely to be missing from the panel part of the sample than those without limitations. This direction of the bias would be expected in the presence of health-related attrition.

As indicated in the graphs, the mean difference across country pairs of HLY is close to zero and thus negligible. Moreover, most of the country-period observations (grey dots) lie within the 95% LoA (dotted lines). Thus, the plots indicate a good agreement between HLYs based on total and rotational sub-samples. The 5–6% of observations that lie outside the LoA are almost equally distributed above and below the intervals. In most cases, we observe that only a single observation per country is outside the LoA, so most of the outliers appear to be random. For countries with repeated observations outside of the LoA (e.g. Greece in 2018 and 2019 in all plots), outliers can mainly be attributed to overlapping sub-samples. The repeated error in overlapping samples indicates a problem with the representativeness of the sub-samples and not necessarily selective panel attrition. In contrast, a systematic bias is observed in Slovakia, where, for both sexes, we observe three independent samples (2009, 2013 and 2018) with differences between HLYs at ages 50 and 65 years outside of the LoA. We also observe differences outside of the LoA for HLYs at birth in Latvia (both sexes, 2008 and 2019) and Luxembourg (men only, 2013 and 2017).

The LoA represent an interval for the measurement’s random error related to sample attrition. With up to ±2.1 years at birth, we find that the LoA are large, reflecting a high level of uncertainty; the LoA are wider for women than for men. While in absolute terms, the LoA are largest for the difference in HLYs at birth, in relative terms, the uncertainty increases with age, amounting to up to ±14.4% at age 65.

The LoA are wider than the CIs of the HLY estimates, hence indicating that attrition alone leads to greater uncertainty in the point estimates than is accounted for in the CIs estimated of HLYs based on the total sample. The CIs of HLYs are up to ±1.6 and 1.7 years at birth; 0.8 and 1 year at age 50; 0.7 and 1 year at age 65, for men and women, respectively (individual values in the Supplementary materials).

## Discussion

We find that across countries, HLYs reported by Eurostat to monitor the population health of Europeans are not significantly biased by health-related attrition. However, sample attrition increases the uncertainty in the measurement of HLYs of individual countries, as indicated by the LoA being wider than the CIs of HLYs. Consequently, health-related attrition should be acknowledged in cross-sectional estimates of HLY based on longitudinal samples of EU-SILC.

We also find a systematic bias reflected in repeated outliers across independent samples for a few individual countries: Slovakia, Latvia and Luxembourg.

HLYs is only one of the Eurostat’s statistics on income, social inclusion and living conditions of Europeans based on EU-SILC cross-sectional samples that is likely to be biased by the attrition of the longitudinal sample of the survey (figure 1).

## Supplementary Material

ckad069_Supplementary_Data
