# Supplementary material for: The effect of sample attrition in the EU Statistics on Income and Living Conditions on the estimates of Eurostat’s Healthy Life Years
Source: Eur J Public Health. 2023 Apr 24;33(3):378–80. doi: 10.1093/eurpub/ckad069 (PMC10234637; doi:10.1093/eurpub/ckad069)
Supplement: ckad069_Supplementary_Data [file ckad069_supplementary_data.zip › ckad069_Supplementary_Data/ejph-2022-02-sr-0107-File003.pdf]

1 Bland-Altman plots

Following the guidelines developed by Kottner et al. (2011) for reporting reliability and agreement studies, Bland-Altman plots with limits of agreement are a recommended method for analysing the agreement of continuous variables.
In Bland-Altman plots the difference between two variables for each observation point is plotted against the mean. If the differences are normally distributed, $(1-\alpha)\%$ of the observations will lie between the limits of agreement of $d - \alpha s$ and $d + \alpha s$ where d is the mean and s is the standard deviation of differences between variables, and z_α is the upper $1-\alpha/2$ critical value for the standard normal distribution (1).

2 Supplementary Tables

Table 1: The assigned number to the new rotational samples in cross-sectional EU-SILC datasets in each year by country

Country	2007	2008	2009	2010	2011	2012	2013	2014	2015	2016	2017	2018	2019
AT	3	4	1	2	3	4	1	2	3	4	1	2	3
BE	3	4	1	2	3	4	1	2	3	4	1	2	5
BG	-	-	3	4	1	2	3	4	1	6	5	2	3
CH	-	1	2	3	4	1	2	3	4	1	2	3	4
CY	-	3	4	1	2	3	4	1	2	3	4	1	2
CZ	-	-	-	-	-	-	-	2	3	4	1	2	3
DE	-	-	-	1	2	3	4	1	2	3	4	1	2
DK	4	1	2	3	4	1	2	3	4	1	2	3	4
EE	-	-	1	2	3	4	1	2	3	4	1	2	3
EL	-	-	-	-	-	1	2	3	4	1	2	3	4
ES	3	4	1	2	3	4	1	2	3	4	1	2	3
FI	-	-	-	1	2	3	4	1	2	3	4	1	2
FR	-	4	5	6	7	8	9	1	2	3	4	5	6
HR	-	-	-	1	4	1	3	2	4	1	3	2	4
IT	3	4	1	2	3	4	1	2	3	4	1	2	3
LT	2	3	4	1	2	3	4	1	2	3	4	1	2
LU	-	-	-	-	-	-	4	1	2	3	4	1	2
LV	2	3	4	1	2	3	4	1	2	3	4	1	2
MT	3	4	2	1	3	4	2	1	3	4	2	1	3
NL	2	3	4	1	2	3	4	1	2	3	4	1	2
NO	-	-	-	-	-	-	-	-	1	2	3	4	1
PL	2	3	4	1	2	3	4	1	2	3	4	1	2
PT	-	4	1	2	3	4	1	2	3	4	1	2	3
RO	4	1	2	3	4	1	2	3	4	1	2	3	4
RS	-	-	-	-	-	-	3	1	2	3	2	1	2
SI	1	2	3	4	1	2	3	4	1	2	3	4	1
SK	2	3	4	1	2	3	4	1	2	3	4	1	2
UK	2	3	4	1	2	3	4	1	2	3	4	1	-

Note: when not available, most likely we could not recognize from the official documentation, which sub-sample is the new rotational one; Source: Collected from the official documentation of EU-SILC available at https://circabc.europa.eu under Eurostat/EU-SILC/quality$_{a}ssesment/quality_{r}eports$

3 Additional files

Files "totalsampleHLY.csv" and "newrotationalHLY.csv" in the Supplementary Material contain Healthy Life Years at birth, age 50 and 65 years, based on total cross-sectional samples and new rotational samples, by sex and country, 2007-2019.
File ""HLYCI.csv" in the Supplementary Material contains confidence intervals for Healthy Life Years at birth, age 50 and 65 years, for total cross-sectional samples, by sex and country, 2007-2019.

References

[1] Bland JM, Altman D. Statistical methods for assessing agreement between two methods of clinical measurement. The lancet. 1986;327(8476):307-10.
